# Nepal's War and Conflict-Sensitive Development

**DOI:** 10.1371/journal.pmed.0020029

**Published:** 2005-01-25

**Authors:** Sonal Singh

**Affiliations:** Unity Health SystemRochester, New YorkUnited States of America

I would like to share my experience from nearly a decade of civil war between the Maoist rebels and the Royal Nepalese Army in Nepal in reference to the article by Zwi [1] on the expanding role of health communities in times of conflict. The current war in Nepal has led to widespread destruction of limited infrastructure and has adversely impacted access to health-care services and personnel, affecting family planning, maternal and child health programs, and immunization services throughout the country. While Nepal is flooded with non-governmental organizations, paradoxically, humanitarian assistance may have unknowingly exacerbated the conflict by perpetuating the same inequalities that led to the conflict in the first place. This has brought to the fore the need for “conflict-sensitive development” [2]—development sensitive to the (conflict) environments in which they operate, in order to reduce the negative impacts of their activities—and to increase their positive impacts—on the situation and its dynamics. Development projects can continue in less affected areas with a need for transitional programs in conflict areas that can adapt to the rapidly changing environment. If agencies are unable to function, they have required the help of humanitarian agencies such as Médicins Sans Frontières with experience in conflict settings. Some agencies have adopted a participatory role in development and have involved neutral local agencies, increasing community participation in their projects with good success. But there is a need for increasing coordination between organizations working in various health-related projects. Health-care workers across the world in different conflicts are in a unique position to leverage something of universal importance—the promise of good health [3]. Raising awareness of the issues surrounding conflicts will act as a catalyst for change.
